# Species distribution and antimicrobial resistance of *Staphylococcus* spp. isolated from dogs and cats in Poland: a retrospective laboratory-based study

**DOI:** 10.3389/fvets.2026.1857011

**Published:** 2026-05-21

**Authors:** Jan Tyc, Dawid Jańczak, Piotr Górecki, Weronika Wójtowicz, Katarzyna Hylińska, Grzegorz Woźniakowski

**Affiliations:** 1 Department of Infectious, Invasive Diseases and Veterinary Administration, Institute of Veterinary Medicine, Faculty of Biological and Veterinary Sciences, Nicolaus Copernicus University, Toruń, Poland; 2 Animallab Veterinary Laboratory, Warsaw, Poland; 3 Animallab Veterinary Laboratory, Łódź, Poland

**Keywords:** antimicrobial resistance (AMR), cats, dogs, MIC, multidrug resistance (MDR), *Staphylococcus felis*, *Staphylococcus pseudintermedius*

## Abstract

**Introduction:**

*Staphylococcus* spp. are important opportunistic pathogens of companion animals and an increasingly relevant component of the antimicrobial resistance problem in veterinary medicine. The aim of this study was to evaluate the species distribution and antimicrobial susceptibility patterns of *Staphylococcus* spp. isolated from dogs and cats in Poland, with particular emphasis on MIC-based testing.

**Methods:**

A total of 281 records of *Staphylococcus* isolates obtained from routine diagnostic submissions to a commercial veterinary laboratory were included. Bacterial identification was performed using MALDI-TOF-MS, and antimicrobial susceptibility was assessed using a commercial MIC panel.

**Results:**

After exclusion of nitrofurantoin from comparative analyses, the highest resistance rates were observed for penicillin (86.5%), trimethoprim-sulfamethoxazole (77.2%), and tetracycline (68.0%), whereas no resistant isolates were detected for teicoplanin or vancomycin. Direct MIC-based interpretation of ampicillin-sulbactam yielded no resistant isolates. *Staphylococcus pseudintermedius* was the predominant species in dogs, while *Staphylococcus felis* predominated in cats. Dog-derived isolates showed a significantly higher resistance burden than cat-derived isolates, and multidrug resistance was significantly more frequent in isolates obtained from dogs than cats (72.5% vs. 55.7%). Among the four dominant species, resistance burden and multidrug resistance rates were highest in *S. pseudintermedius*. Significant between-host differences after multiple-testing correction were retained for erythromycin and trimethoprim-sulfamethoxazole, both being more frequent in canine isolates.

**Discussion:**

Companion animal staphylococci in Poland exhibit substantial resistance to several commonly used antimicrobials, with *S. pseudintermedius* representing the major driver of resistance in dogs. These findings highlight the need for continued surveillance and prudent antimicrobial stewardship in small animal practice.

## 1 Introduction

Antimicrobial resistance (AMR) is recognized as one of the most important contemporary threats to human and animal health. Its global influence is not limited to therapeutic failure. It also contributes to an overall increase in morbidity, mortality, and healthcare burden (1, 2). Due to their clinical significance, their ability to colonize skin and mucosal surfaces, and their exceptional genetic plasticity—which facilitates the acquisition and spread of resistance determinants through mobile genetic elements—staphylococci stand out among Gram-positive bacteria (3, 4). As a result, the issue of antimicrobial resistance in staphylococci should be viewed as part of a more comprehensive One Health framework, rather than as a problem that is exclusive to human or veterinary medicine (5, 6).

In companion animals, staphylococci are an important component of the normal microbiota of the skin and mucous membranes; nonetheless, they may also act as opportunistic pathogens. In dogs, *Staphylococcus pseudintermedius* is considered the predominant staphylococcal species, whereas in cats, *Staphylococcus felis* is commonly reported as a common colonizer (7, 8). Under conditions such as atopy, immunosuppression, or disruption of local barriers, these bacteria may be involved in numerous infections, including pyoderma, otitis externa, conjunctivitis, pododermatitis, and urinary tract infections (7, 9, 10). Therefore, the transition of staphylococci from commensal colonizers to clinically relevant pathogens is of major importance in small animal practice.

The epidemiological significance of veterinary staphylococci is further reinforced by their zoonotic potential. Although some species, such as *S. pseudintermedius* and *S. felis*, are considered primarily host-associated, transmission from companion animals to humans has been already documented (11, 12). Evidence suggests that the exchange of microbial populations between humans, animals, and their common environments over time may improve the circulation of resistant strains and resistance genes, rendering this issue more significant (6). Several studies have already evaluated the prevalence and epidemiology of staphylococci in dogs and cats, including surveys carried out in Poland (8, 13–15). These reports confirmed the importance of *S. pseudintermedius* in dogs and demonstrated that *S. felis* and *S. pseudintermedius* are also relevant components of the feline staphylococcal population (8, 13, 15). In addition, recent Polish data have emphasized the therapeutic challenges associated with resistant canine staphylococci, particularly methicillin-resistant *S. pseudintermedius* (14). Nevertheless, although several recent studies have addressed species-level comparisons and MIC-based resistance in companion animals (16, 17), available data remain limited, especially with regard to broad species-level comparisons of both coagulase-positive staphylococci (CoPS) and coagulase-negative staphylococci (CoNS) from dogs and cats, as well as susceptibility testing based on minimum inhibitory concentration (MIC), which is considered the reference approach in clinical microbiology.

Continuous monitoring of antimicrobial susceptibility is necessary due to the clinical significance of staphylococci in companion animals and the dynamic nature of resistance trends. Therefore, the objective of the current report was to assess antimicrobial resistance patterns and species distribution of coagulase-positive and coagulase-negative staphylococci that were isolated from dogs and cats in Poland in 2024, with a particular focus on MIC-based susceptibility testing.

## 2 Materials and methods

### 2.1 Study population characteristics

The dataset comprised 281 *Staphylococcus* isolate records obtained from dogs and cats, including 211 dog-derived isolates (75.1%) and 70 cat-derived isolates (24.9%). Overall, 169/281 (60.1%) records originated from male animals and 112/281 (39.9%) from female animals (dogs: 126 males and 85 females; cats: 43 males and 27 females). The odds of a record originating from a male animal did not differ significantly between dogs and cats (OR 0.93, 95% CI 0.53–1.62). Age data were available for 258/281 (91.8%) records, and the median age was 5.8 years (IQR 2.5–9.0), ranging from 3 months to 16 years.

### 2.2 Specimen collection

The 281 *Staphylococcus* spp. isolates originated from a retrospective diagnostic cohort of clinical specimens submitted in 2024 by external veterinary clinics to a commercial laboratories (Animallab Veterinary Laboratory, Warsaw and Łódź, Poland). Clinical specimens included skin swabs (104/281; 37.0%), external ear canal swabs (79/281; 28.1%), nasal cavity swabs (42/281; 14.9%), wound swabs (24/281; 8.5%), conjunctival sac swabs (16/281; 5.7%), and the urogenital tract swabs (16/281; 5.7%). All bacteriological swabs were collected for routine clinical purposes and submitted as part of standard diagnostic work-up, and only anonymized metadata were used for analysis.

### 2.3 Bacterial culture and antimicrobial susceptibility testing

Swabs were inoculated onto 5% defibrinated sheep blood agar and MacConkey agar plates (GRASO Biotech, Starogard Gdański, Poland). The plates were incubated under aerobic and microaerophilic conditions at 35 C± 2 °C for 24, 48, and 72 h. Bacterial identification was performed using the MALDI Biotyper® system, with spectra acquisition and analysis conducted using MBT Compass version 4.1 software (Bruker Daltonics GmbH & Co. KG, Bremen, Germany).

Antimicrobial susceptibility was evaluated by the broth microdilution method using a commercial MIC panel (DIAGNOSTICS s.r.o., Galanta, Slovakia), which included the following antimicrobials: penicillin, ampicillin-sulbactam, gentamicin, erythromycin, clindamycin, chloramphenicol, ciprofloxacin, tetracycline, nitrofurantoin, trimethoprim-sulfamethoxazole, teicoplanin, and vancomycin. MIC results were interpreted primarily according to CLSI VET01S for bacteria isolated from animals (18). When veterinary-specific MIC breakpoints were not publicly identifiable for the exact antimicrobial-agent/organism combination, human CLSI M100 surrogate criteria were applied (19). In particular, archived CLSI M100 breakpoints for *Staphylococcus* spp. were used for ampicillin-sulbactam, whereas detailed MIC interpretive criteria applied in this study are summarized in Table 1. Nitrofurantoin was included in the testing panel but was excluded from comparative statistical analyses because the isolates were not of UTI origin.

**Table 1:** MIC interpretive criteria applied in this study for *Staphylococcus* spp. isolated from dogs and cats.

Antimicrobial	Breakpoint applied in this study	Susceptible (S), μg/mL	Intermediate (I), μg/mL	Resistant (R), μg/mL	Primary interpretive basis
PEN	*Staphylococcus* spp.	≤0.12	–	≥0.25	CLSI M100
AMS	*Staphylococcus* spp.	≤8/4	16/8	≥32/16	CLSI M100
GEN	*Staphylococcus* spp.	≤4	8	≥16	CLSI M100
ERY	*Staphylococcus* spp.	≤0.5	1–4	≥8	CLSI M100
CLI	*Staphylococcus* spp.	≤0.5	1–2	≥4	CLSI M100
CHL	*Staphylococcus* spp.	≤2	4	≥8	CLSI VET01S
CIP	*Staphylococcus* spp.	≤1	2	≥4	CLSI M100
TET	*Staphylococcus* spp.	≤0.25	0.5	≥1	CLSI VET01S
NFE	Urinary *Staphylococcus* isolates only	≤32	64	≥128	CLSI M100
STX	*Staphylococcus* spp.	≤2/38	–	≥4/76	CLSI M100
TEI^a^	*S. aureus*; CoNS/non-aureus staphylococci	≤8	16	≥32	CLSI M100
VAN	*S. aureus*	≤2	4–8	≥16	CLSI M100
VAN	Other *Staphylococcus* spp. / coagulase-negative staphylococci	≤4	8–16	≥32	CLSI M100

PEN, penicillin; AMS, ampicillin-sulbactam; GEN, gentamicin; ERY, erythromycin; CLI, clindamycin; CHL, chloramphenicol; CIP, ciprofloxacin; TET, tetracycline; STX, trimethoprim-sulfamethoxazole; TEI, teicoplanin; VAN, vancomycin.

a Teicoplanin was retained only as a historical human CLSI-derived surrogate (*S* ≤ 8, *I* 16, *R* ≥ 32) and should be treated as supportive rather than a primary veterinary interpretive criterion.

### 2.4 Statistical analysis

Antimicrobial susceptibility results were categorized as susceptible (S), intermediate (I), or resistant (R) according to the MIC interpretive criteria adopted for this study. For each isolate, the resistance burden was defined as the number of antimicrobial agents categorized as resistant. Multidrug resistance (MDR) was defined according to Magiorakos et al. (20) as non-susceptibility to at least one agent in ≥3 antimicrobial classes. The following classes were considered: beta-lactams, aminoglycosides, macrolides, lincosamides, phenicols, fluoroquinolones, tetracyclines, folate pathway inhibitors, and glycopeptides. Isolates with missing age data were excluded from age-based analyses and from the multivariable model. Descriptive data are presented as counts and percentages for categorical variables and as medians with interquartile ranges (IQRs) for non-normally distributed continuous variables. The association between age and resistance burden was assessed using Spearman’s rank correlation coefficient. Comparisons of resistance burden between two groups, including sex and animal species, were performed using the Mann–Whitney U test. Differences in resistance burden among the four main *Staphylococcus* species (*S. pseudintermedius, S. felis, S. aureus,* and *S. intermedius*) were evaluated using the Kruskal–Wallis test, followed by pairwise *post hoc* comparisons with Holm correction. The association between animal species and the distribution of cultured *Staphylococcus* species was assessed using the chi-square test.

For the extended analysis, MDR was compared between dogs and cats and between sexes using Fisher’s exact test, where MDR frequencies among the four main *Staphylococcus* species were compared using the chi-square test, followed by pairwise Fisher’s exact tests with Holm correction. For each antimicrobial agent, the resistance was dichotomized as R versus non-R (S + I). The frequency of R versus non-R was compared between dogs and cats using Fisher’s exact test, and between the four main *Staphylococcus* species using the chi-square test. Holm correction was applied separately within the host-species antimicrobial panel and within the species-level antimicrobial panel. A multivariable logistic regression model was constructed for MDR, with animal species, sex, age, and cultured *Staphylococcus* species included as predictors. Rare *Staphylococcus* species were pooled into an “Other” category. The model was fitted only on complete cases. Statistical analyses were performed using R version 4.5.3 (R Foundation for Statistical Computing, Vienna, Austria). A two-sided *p*-value of <0.05 was considered statistically significant.

## 3 Results

After exclusion of nitrofurantoin, antimicrobial susceptibility was interpreted for 281 staphylococcal isolates. The highest resistance rates were observed for penicillin (243/281, 86.5%), trimethoprim-sulfamethoxazole (217/281, 77.2%), and tetracycline (191/281, 68.0%). In contrast, no resistant isolates were detected for teicoplanin or vancomycin. Direct MIC-based interpretation of ampicillin-sulbactam showed no resistant isolates; 250/281 (89.0%) isolates were assumed as susceptible and 31/281 (11.0%) as intermediate (Table 2).

**Table 2:** Overall antimicrobial susceptibility categories for all isolates (*n* = 281).

Antimicrobial	*S*, *n* (%)	*I*, *n* (%)	*R*, *n* (%)
PEN	38 (13.5%)	0 (0.0%)	243 (86.5%)
AMS	250 (89.0%)	31 (11.0%)	0 (0.0%)
GEN	230 (81.9%)	11 (3.9%)	40 (14.2%)
ERY	135 (48.0%)	23 (8.2%)	123 (43.8%)
CLI	151 (53.7%)	14 (5.0%)	116 (41.3%)
CHL	112 (39.9%)	72 (25.6%)	97 (34.5%)
CIP	169 (60.1%)	27 (9.6%)	85 (30.2%)
TET	48 (17.1%)	42 (14.9%)	191 (68.0%)
STX	64 (22.8%)	0 (0.0%)	217 (77.2%)
TEI	275 (97.9%)	6 (2.1%)	0 (0.0%)
VAN	276 (98.2%)	5 (1.8%)	0 (0.0%)

PEN, penicillin; AMS, ampicillin-sulbactam; GEN, gentamicin; ERY, erythromycin; CLI, clindamycin; CHL, chloramphenicol; CIP, ciprofloxacin; TET, tetracycline; STX, trimethoprim-sulfamethoxazole; TEI, teicoplanin; VAN, vancomycin.

**Figure 1:**
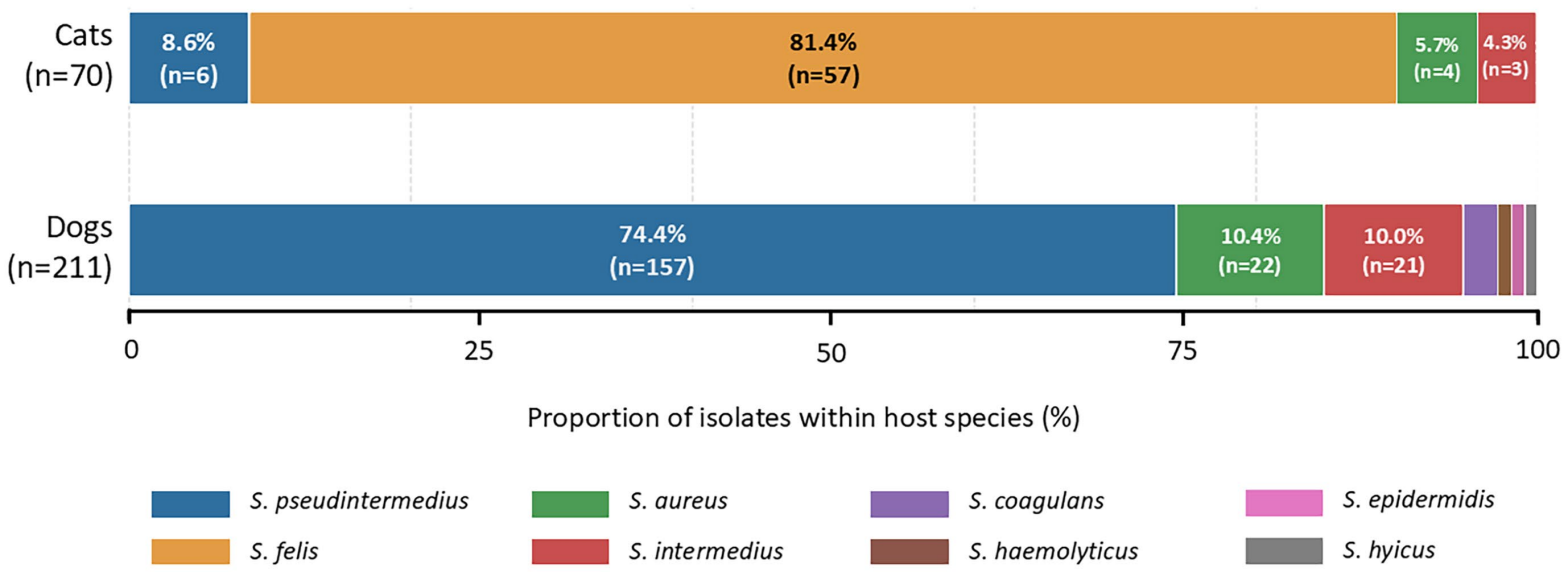
Distribution of *Staphylococcus* species among dog- and cat-derived isolates. Bars show the proportion of each *Staphylococcus* species within host species. *S. pseudintermedius* predominated among dog-derived isolates, whereas *S. felis* was the dominant species among cat-derived isolates.

The overall resistance burden had a median of 4 resistant antimicrobial agents per isolate (IQR 2–6). No significant correlation was found between age and resistance burden (Spearman’s rho = 0.093, *p* = 0.135), and no significant difference was observed between male and female animals (Mann–Whitney *U* = 10,284.5, *p* = 0.214). In contrast, isolates obtained from dogs showed a significantly higher resistance burden than isolates obtained from cats (median 4 [IQR 2–6] vs. 3 [IQR 2–5], Mann–Whitney *U* = 9,052.5, *p* = 0.004). The distribution of cultured *Staphylococcus* species differed markedly between dogs and cats (chi-square = 217.98, df = 7, *p* < 0.001), with *S. pseudintermedius* predominating in dogs and *S. felis* in cats (Figure 1; Table 3).

**Table 3:** Resistance rates by animal species.

Antimicrobial	Dogs (*n* = 211)	Cats (*n* = 70)
PEN	187/211 (88.6%)	56/70 (80.0%)
AMS	0/211 (0.0%)	0/70 (0.0%)
GEN	30/211 (14.2%)	10/70 (14.3%)
ERY	104/211 (49.3%)	19/70 (27.1%)
CLI	96/211 (45.5%)	20/70 (28.6%)
CHL	78/211 (37.0%)	19/70 (27.1%)
CIP	67/211 (31.8%)	18/70 (25.7%)
TET	143/211 (67.8%)	48/70 (68.6%)
STX	173/211 (82.0%)	44/70 (62.9%)
TEI	0/211 (0.0%)	0/70 (0.0%)
VAN	0/211 (0.0%)	0/70 (0.0%)

PEN, penicillin; AMS, ampicillin-sulbactam; GEN, gentamicin; ERY, erythromycin; CLI, clindamycin; CHL, chloramphenicol; CIP, ciprofloxacin; TET, tetracycline; STX, trimethoprim-sulfamethoxazole; TEI, teicoplanin; VAN, vancomycin.

Statistical comparisons between dogs and cats were performed using Fisher’s exact test; detailed results are presented in Table 6.

**Figure 2:**
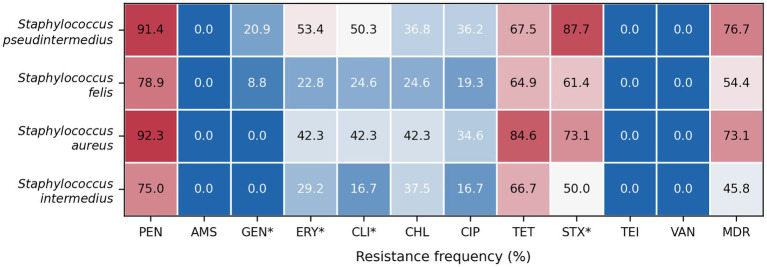
Heatmap of antimicrobial resistance frequencies (%) among the four predominant *Staphylococcus* species isolated from dogs and cats. Values represent the percentage of isolates classified as resistant (R) according to MIC-based interpretation. Multidrug resistance (MDR) is defined as resistance to at least one antimicrobial agent in ≥3 antimicrobial classes. Asterisks (*) indicate statistically significant differences between species after Holm correction (*p* < 0.05). Color intensity reflects resistance frequency, with higher values indicated by warmer colors. PEN, penicillin; AMS, ampicillin-sulbactam; GEN, gentamicin; ERY, erythromycin; CLI, clindamycin; CHL, chloramphenicol; CIP, ciprofloxacin; TET, tetracycline; STX, trimethoprim-sulfamethoxazole; TEI, teicoplanin; VAN, vancomycin.

The four main *Staphylococcus* species identified in the study were *S. pseudintermedius* (163/281, 58.0%), *S. felis* (57/281, 20.3%), *S. aureus* (26/281, 9.3%), and *S. intermedius* (24/281, 8.5%). Resistance burden differed significantly among these four species (Kruskal–Wallis H = 24.48, *p* < 0.001). In pairwise *post hoc* comparisons with Holm correction, *S. pseudintermedius* isolates were significantly more resistant than *S. felis* (adjusted *p* = 0.00016) and *S. intermedius* (adjusted *p* = 0.006), whereas the remaining pairwise comparisons were not statistically significant. Species-specific resistance profiles for the four dominant *Staphylococcus* species are shown in Figure 2 and Table 4.

**Table 4:** Resistance rates in the four main *Staphylococcus* species.

Antimicrobial	*S. pseudintermedius* (*n* = 163)	*S. felis* (*n* = 57)	*S. aureus* (*n* = 26)	*S. intermedius* (*n* = 24)
PEN	149/163 (91.4%)	45/57 (78.9%)	24/26 (92.3%)	18/24 (75.0%)
AMS	0/163 (0.0%)	0/57 (0.0%)	0/26 (0.0%)	0/24 (0.0%)
GEN	34/163 (20.9%)	5/57 (8.8%)	0/26 (0.0%)	0/24 (0.0%)
ERY	87/163 (53.4%)	13/57 (22.8%)	11/26 (42.3%)	7/24 (29.2%)
CLI	82/163 (50.3%)	14/57 (24.6%)	11/26 (42.3%)	4/24 (16.7%)
CHL	60/163 (36.8%)	14/57 (24.6%)	11/26 (42.3%)	9/24 (37.5%)
CIP	59/163 (36.2%)	11/57 (19.3%)	9/26 (34.6%)	4/24 (16.7%)
TET	110/163 (67.5%)	37/57 (64.9%)	22/26 (84.6%)	16/24 (66.7%)
STX	143/163 (87.7%)	35/57 (61.4%)	19/26 (73.1%)	12/24 (50.0%)
TEI	0/163 (0.0%)	0/57 (0.0%)	0/26 (0.0%)	0/24 (0.0%)
VAN	0/163 (0.0%)	0/57 (0.0%)	0/26 (0.0%)	0/24 (0.0%)

PEN, penicillin; AMS, ampicillin-sulbactam; GEN, gentamicin; ERY, erythromycin; CLI, clindamycin; CHL, chloramphenicol; CIP, ciprofloxacin; TET, tetracycline; STX, trimethoprim-sulfamethoxazole; TEI, teicoplanin; VAN, vancomycin.

Overall comparisons across species were performed using the chi-square test with Holm correction; detailed results of the statistical analysis are presented in Table 7.

MDR, defined as resistance to at least one antimicrobial in at least three antimicrobial classes, was identified in 192/281 isolates (68.3%). MDR was significantly more frequent among canine than feline isolates (153/211, 72.5% vs. 39/70, 55.7%; OR = 2.10, Fisher’s exact *p* = 0.0116), whereas no significant association was observed with sex (male: 119/169, 70.4% vs. female: 73/112, 65.2%; OR = 1.27, *p* = 0.363). Among the four main *Staphylococcus* species, MDR rates differed significantly overall (chi-square = 16.38, df = 3, *p* = 0.00095), with the highest proportions observed in *S. pseudintermedius* (76.7%) and *S. aureus* (73.1%), compared with *S. felis* (54.4%) and *S. intermedius* (45.8%). In pairwise *post hoc* analyses with Holm correction, *S. pseudintermedius* showed significantly higher MDR rates than *S. felis* (adjusted *p* = 0.013) and *S. intermedius* (adjusted *p* = 0.014) (Table 5).

**Table 5:** Multidrug resistance (MDR) in the study population and multivariable logistic regression for MDR.

A. Univariable MDR comparisons			
Variable	Category	MDR, *n*/*N* (%)	Statistical result
Animal species	Dog	153/211 (72.5%)	OR = 2.10 vs. cats; Fisher’s exact *p* = 0.0116
	Cat	39/70 (55.7%)	Reference
Sex	Male	119/169 (70.4%)	OR = 1.27 vs. females; Fisher’s exact *p* = 0.3626
	Female	73/112 (65.2%)	Reference
Main *Staphylococcus* species	*S. pseudintermedius*	125/163 (76.7%)	Overall chi-square = 16.38, df = 3, *p* = 0.00095
	*S. felis*	31/57 (54.4%)	
	*S. aureus*	19/26 (73.1%)	
	*S. intermedius*	11/24 (45.8%)	
B. Multivariable logistic regression model for MDR			
Predictor	OR	95% CI	*p-*value
Dog vs. cat	1.79	0.51–6.33	0.3675
Male vs. female	1.26	0.72–2.20	0.4206
*S. pseudintermedius* vs. *S. felis*	1.70	0.44–6.62	0.4420
*S. aureus* vs. *S. felis*	1.25	0.28–5.55	0.7732
*S. intermedius* vs. *S. felis*	0.43	0.10–1.89	0.2629
Other *Staphylococcus* spp. vs. *S. felis*	0.39	0.06–2.64	0.3376
Age (per 12-month increase)	0.96	0.89–1.03	0.2648

MDR was defined as resistance to at least one antimicrobial in at least three antimicrobial classes. In post hoc pairwise comparisons with Holm correction, *S. pseudintermedius* had significantly higher MDR rates than *S. felis* (adjusted *p* = 0.013) and *S. intermedius* (adjusted *p* = 0.014). The logistic regression model was fitted on 258 complete cases; rare species were pooled into the “Other” category.

When individual antimicrobials were analyzed as R versus non-R, significant differences between dogs and cats after Holm correction were retained for erythromycin (49.3% vs. 27.1%, adjusted *p* = 0.0106) and trimethoprim-sulfamethoxazole (82.0% vs. 62.9%, adjusted *p* = 0.0113), both being more common in canine isolates (Table 6). Across the four main *Staphylococcus* species, overall resistance frequencies differed significantly for gentamicin (adjusted *p* = 0.0077), erythromycin (adjusted *p* = 0.0026), clindamycin (adjusted *p* = 0.0026), and trimethoprim-sulfamethoxazole (adjusted *p* < 0.001) (Table 7). *Post hoc* comparisons indicated that *S. pseudintermedius* was the main driver of these differences. A multivariable logistic regression model for MDR, fitted on 258 complete cases, was significant overall (likelihood ratio test *p* = 0.008; pseudo-*R*^2^ = 0.059), but none of the individual predictors remained statistically significant in the adjusted model (Table 5).

**Table 6:** Comparison of resistance frequencies (R vs. non-R) between dog and cat isolates.

Antimicrobial	Dogs, R *n*/*N* (%)	Cats, R *n*/*N* (%)	OR (Dogs vs. Cats)	*p-*value	Holm-adjusted *p*
PEN	187/211 (88.6%)	56/70 (80.0%)	1.95	0.0727	0.3637
AMS	0/211 (0.0%)	0/70 (0.0%)	—	—	—
GEN	30/211 (14.2%)	10/70 (14.3%)	0.99	1.0000	1.0000
ERY	104/211 (49.3%)	19/70 (27.1%)	2.61	0.0013	0.0106
CLI	96/211 (45.5%)	20/70 (28.6%)	2.09	0.0169	0.1012
CHL	78/211 (37.0%)	19/70 (27.1%)	1.57	0.1487	0.5946
CIP	67/211 (31.8%)	18/70 (25.7%)	1.34	0.3710	1.0000
TET	143/211 (67.8%)	48/70 (68.6%)	0.96	1.0000	1.0000
STX	173/211 (82.0%)	44/70 (62.9%)	2.69	0.0016	0.0113
TEI	0/211 (0.0%)	0/70 (0.0%)	—	—	—
VAN	0/211 (0.0%)	0/70 (0.0%)	—	—	—

PEN, penicillin; AMS, ampicillin-sulbactam; GEN, gentamicin; ERY, erythromycin; CLI, clindamycin; CHL, chloramphenicol; CIP, ciprofloxacin; TET, tetracycline; STX, trimethoprim-sulfamethoxazole; TEI, teicoplanin; VAN, vancomycin.

Resistance was dichotomized as R versus non-R, where non-R included susceptible and intermediate isolates. Fisher’s exact test was used for all comparisons. Holm correction was applied across the full antimicrobial panel.

**Table 7:** Comparison of resistance frequencies (R vs. non-R) among the four main *Staphylococcus* species.

Antimicrobial	*S. pseudintermedius n*/*N* (%)	*S. felis n*/*N* (%)	*S. aureus n*/*N* (%)	*S. intermedius n*/*N* (%)	Overall *p-*value	Holm-adjusted *p*
PEN	149/163 (91.4%)	45/57 (78.9%)	24/26 (92.3%)	18/24 (75.0%)	0.0185	0.0741
AMS	0/163 (0.0%)	0/57 (0.0%)	0/26 (0.0%)	0/24 (0.0%)	—	—
GEN	34/163 (20.9%)	5/57 (8.8%)	0/26 (0.0%)	0/24 (0.0%)	0.0015	0.0077
ERY	87/163 (53.4%)	13/57 (22.8%)	11/26 (42.3%)	7/24 (29.2%)	<0.001	0.0026
CLI	82/163 (50.3%)	14/57 (24.6%)	11/26 (42.3%)	4/24 (16.7%)	<0.001	0.0026
CHL	60/163 (36.8%)	14/57 (24.6%)	11/26 (42.3%)	9/24 (37.5%)	0.3023	0.6047
CIP	59/163 (36.2%)	11/57 (19.3%)	9/26 (34.6%)	4/24 (16.7%)	0.0421	0.1262
TET	110/163 (67.5%)	37/57 (64.9%)	22/26 (84.6%)	16/24 (66.7%)	0.3098	0.6047
STX	143/163 (87.7%)	35/57 (61.4%)	19/26 (73.1%)	12/24 (50.0%)	<0.001	<0.001
TEI	0/163 (0.0%)	0/57 (0.0%)	0/26 (0.0%)	0/24 (0.0%)	—	—
VAN	0/163 (0.0%)	0/57 (0.0%)	0/26 (0.0%)	0/24 (0.0%)	—	—

PEN, penicillin; AMS, ampicillin-sulbactam; GEN, gentamicin; ERY, erythromycin; CLI, clindamycin; CHL, chloramphenicol; CIP, ciprofloxacin; TET, tetracycline; STX, trimethoprim-sulfamethoxazole; TEI, teicoplanin; VAN, vancomycin.

Resistance was dichotomized as R versus non-R, where non-R included susceptible and intermediate isolates. Overall comparisons were performed using the chi-square test. Holm correction was applied across the full antimicrobial panel. Significant post hoc pairwise differences were driven mainly by *S. pseudintermedius*, particularly for gentamicin, erythromycin, clindamycin, and trimethoprim-sulfamethoxazole.

## 4 Discussion

The present study provides a laboratory-based overview of the species distribution and MIC-based antimicrobial susceptibility of *Staphylococcus* spp. isolated from dogs and cats in Poland. The most important microbiological finding was the clear host-associated distribution of the dominant species: *S. pseudintermedius* among dog-derived isolates, whereas *S. felis* was the most common species in cats. This pattern is consistent with the established role *of S. pseudintermedius* as the principal opportunistic staphylococcal pathogen in dogs and with previous reports indicating the epidemiological importance of *S. felis* in the feline staphylococcal population (7, 8, 13, 15, 21). Therefore, our data support the view that, despite the inclusion of isolates from several anatomical locations, the staphylococcal population in companion animals remains a strongly host-associated structure.

In the present study, the highest resistance rates were observed for penicillin, trimethoprim-sulfamethoxazole, and tetracycline from the antimicrobial susceptibility perspective. Conversely, no resistant isolates were detected for vancomycin or teicoplanin. In addition, substantial resistance rates were recorded for erythromycin, clindamycin, chloramphenicol, and ciprofloxacin. These findings are broadly in agreement with other recent companion animal studies, in which resistance to beta-lactams and numerous commonly used antimicrobial classes was also frequently reported, particularly among canine isolates (22–24). Although direct cross-study comparisons should be made cautiously because of differences in isolate selection, antimicrobial panels, and interpretive criteria. The overall pattern indicates that resistance to commonly used agents remains significant in veterinary-important staphylococci.

An important result of the present study was the significantly higher resistance burden observed in dog-derived isolates compared with cat-derived isolates, together with the significantly higher prevalence of MDR among isolates obtained from dogs. Similar host-related differences have also been reported in recent surveillance data from other countries, where staphylococci isolated from dogs showed a higher frequency of multidrug resistance than feline isolates (22). In our study, this difference is likely influenced not only by the host species but also by the differential bacterial species distribution between dogs and cats. The multivariable logistic regression model confirms this interpretation, as no single predictor remained independently significant after adjustment, indicating that host species and cultured *Staphylococcus* species were strongly correlated rather than acting as fully independent determinants. The high MDR prevalence observed in dogs (72.5%) is clinically concerning and may reflect frequent antimicrobial exposure in small animal practice. From a One Health perspective, such high MDR rates may increase the risk of transmission of resistant staphylococci between animals and humans, especially in close-contact households. Among the four dominant species, *S. pseudintermedius* showed the highest resistance burden and significantly higher MDR rates than *S. felis* and *S. intermedius*. This observation is clinically relevant because *S. pseudintermedius* is one of the most important bacterial pathogens in canine dermatology, otology, and ophthalmology, and is increasingly recognized as a major reservoir of antimicrobial resistance in small animal practice (14), (23–26). Furthermore, recent research conducted in Europe has underscored the epidemiological complexity of resistant *S. pseudintermedius* populations, which includes significant variation in resistance phenotypes among host species and geographic regions (23, 25). Although methicillin resistance was not directly evaluated in the present study using oxacillin, cefoxitin, or molecular detection of *mec* genes, the phenotypic resistance burden observed in *S. pseudintermedius* strongly supports its major contribution to the AMR problem in companion animals. Nevertheless, it is essential to recognize the significance of coagulase-negative staphylococci. In the present study, *S. felis* was the second most common species overall and the most frequent species in cats, but it indicated a lower resistance burden than *S. pseudintermedius*. However, resistant and MDR *S. felis* isolates were also detected. Recent studies on cats have demonstrated that *S. felis* can be isolated from a wide range of clinical sites and that at least some strains possess resistance traits that are clinically significant (21, 27). Likewise, broader studies of coagulase-negative staphylococci from dogs and cats have demonstrated that these organisms may serve as reservoirs of resistance determinants, including methicillin resistance, and should not be regarded exclusively as contaminants (28), (29). Accordingly, both CoPS and CoNS should be considered in companion animal AMR surveillance.

The interpretation of ampicillin-sulbactam deserves particular caution. In the present study, direct MIC-based interpretation yielded no resistant isolates, with most isolates categorized as susceptible and the remainder as intermediate. However, archived human CLSI surrogate criteria were used for this agent, and no oxacillin/cefoxitin testing or molecular confirmation of methicillin resistance was available (18), (19)). Therefore, the apparent activity of ampicillin-sulbactam in this dataset should not be overinterpreted as evidence against the presence of methicillin-resistant strains. Instead, it should be considered a consequence of the interpretive framework used for this specific antimicrobial agent and organism combination. Therefore, the absence of resistant isolates should be interpreted cautiously and may reflect breakpoint limitations rather than true absence of resistance.

Several limitations should be noted. First off, there was little metadata available, and this was a retrospective analysis based on routine diagnostic submissions from a commercial laboratory. Information on prior antimicrobial treatment, hospitalization history, breed, underlying disease, recurrence of infection, and clinical outcome was not available. In particular, the absence of antimicrobial treatment history limits the ability to interpret resistance patterns in the context of prior exposure. Secondly, because the dataset was made up of isolated records, it was impossible to be certain that each record represented a distinct animal. Importantly, the lack of unique animal identifiers means that multiple isolates may have originated from the same individual (e.g., repeated sampling or multiple anatomical sites). This may have led to overrepresentation of certain resistance phenotypes and species, potentially inflating resistance rates and MDR prevalence. Therefore, the results should be interpreted as isolate-based rather than strictly animal-level estimates. Third, the study relied on phenotypic MIC data and did not include molecular confirmation of resistance determinants or strain typing. Fourth, methicillin resistance was not assessed directly, which restricts the interpretation of beta-lactam resistance mechanisms. Finally, the clinical relevance of some isolates, particularly those recovered from sites where colonization and infection may overlap, must be interpreted with caution. Nevertheless, the study provides a valuable and practical overview of the current species distribution and antimicrobial susceptibility patterns of veterinary staphylococci that have been recovered from dogs and cats in Poland. Additionally, it incorporates clinically relevant data from routine diagnostic practice.

## 5 Conclusion

*Staphylococcus* isolates obtained from dogs and cats in Poland showed a clear host-associated species distribution, with *S. pseudintermedius* predominating in dogs and *S. felis* in cats. High resistance rates to penicillin, trimethoprim-sulfamethoxazole, and tetracycline, together with the high prevalence of multidrug resistance, indicate that antimicrobial resistance remains a substantial concern in companion-animal staphylococci. Canine isolates showed a significantly higher resistance burden and higher MDR frequency than feline isolates, and *S. pseudintermedius* emerged as the principal driver of resistance differences among the dominant species. These findings support the need for continued MIC-based surveillance, prudent antimicrobial use, and more detailed future studies incorporating methicillin-specific testing, molecular resistance markers, and richer clinical metadata.

## Data Availability

The original contributions presented in the study are included in the article/supplementary material, further inquiries can be directed to the corresponding author.
